# Development and psychometric testing of the Thai impact and burden of care scale for caregivers of persons with schizophrenia and co-occurring methamphetamine use

**DOI:** 10.12688/f1000research.52288.1

**Published:** 2021-06-17

**Authors:** Ek-uma Imkome

**Affiliations:** 1 Faculty of mental health and psychiatric nursing, Thammasat University, Patuntane, 12120, Thailand

**Keywords:** Schizophrenia, methamphetamine, caregivers, factor analysis, burden care.

## Abstract

**Objective** **:** The objective of the present study was to develop the Thai version of the Impact and Burden of Care Scale for Caregivers of Persons with Schizophrenia and Co-occurring Methamphetamine Use (TIBSCSM) and test its psychometric properties.

**Methods** **: **This instrument development research subjects were 142 caregivers of persons with schizophrenia and co-occurring methamphetamine use. Sample size adequacy was tested by Kaiser–Meyer–Olkin (KMO), and Bartlett’s test of sphericity tested the adequacy of the item correlation matrix. The second-order confirmatory factor analysis (CFA) was used to test the theoretical model.

**Results** The 32-item TIBSCSM showed convergent validity correlations with two quality-of-life measures. Additionally, KMO=0.9, Bartlett’s Test of Sphericity χ2=5248.5, df=496, p<0.001, and internal consistency reliability was high (α=0.9). The CFA has shown that the findings are supported by the theoretical models (χ2=325.2, df=287, p<0.001, RMSEA=0.0, CFI =0.9)

**Conclusion: **The TIBSCSM scale has potential benefits for psychiatric nurses and psychiatric care teams to measure the impact and care burden of caregivers of persons with schizophrenia and methamphetamine use in the areas of nursing, research, education, and clinical determination.

The test results suggested that The TIBSCSM scale has potential benefits for psychiatric and mental health care team to assess the impact and burden care of schizophrenic caregiver for both research and clinical purposes, especially during the COVID-19 pandemic for providing care to relieve the impact and burden of care.

## Introduction

Schizophrenia with co-occurring methamphetamine use is an incapacitating and complex psychiatric illness with either stage, phase, personal factors, and continuous evolution, resulting in impaired physical and psychological health status and social problems. Additionally, it impacts on caregivers’ role and functioning that add to the burden of care and economic consequences. ^1- 3^ Moreover, care burden can lead to a low quality of life (QOL), particularly when suffering significant burdens, resulting in role confusion, poor caring performance, psychosomatic problems, anxiety, stress, and depressive symptoms. The negative emotional experience of caregivers may lead to low performance of caring and have crucial adverse effects on medication compliance, continuity, and a good understanding of the care and social support. ^1, 4^

The caregivers work out ways to take care of persons with schizophrenia and methamphetamine misuse with severe psychotic symptoms to the best of their ability. They try to decrease warning signs such as aggressive and violent behaviors as soon as possible. The caregiver tries to give the reasons, call their name, suggest cool water to drink, or shower and express their love and care by speaking gently. They also deal with hardships in caring for patients, which happen to their belongings, other people, and themselves due to harm resulting from psychotic symptoms. During the relapse phase, caregivers work hard with relapses using many ways to prevent injury, set a safe environment, continue medication adherence and soothe their relatives’ psychological state. Additionally, the caregiver tries to pull their relatives back to a normal state as much as possible by encouraging their relatives’ memories and ability to perform activities of daily living.

Numerous studies of evidence-based practice have illustrated that a lack of primary caregiver involvement in treatment planning is related to the treatment adherence issue. Consequently, evaluating the caregivers’ impact and care burden is of considerable relevance both for caregivers and indirectly for the quality of life of persons with schizophrenia with co-occurring methamphetamine use.

The caregivers’ impact, coping strategies, stress, burden, anxiety, caregiving, and views on the grounds and magnitudes of psychiatric illnesses and co-occurring substance abuse require significant attention. Moreover, currently, the development of psychosocial interventions for caregivers is also a considerable concern. There is an essential need for interventions to enhance caregivers’ emotional health and performance programs delivered by nurses and healthcare teams. ^5^ Based on this, it is necessary to develop good psychometric properties to assess the impact and burden of caregivers of persons with schizophrenia with co-occurring methamphetamine use.

Although studies have been conducted on specific issues in caregiving, little has been done to explore the impact and burden among caregivers of persons with schizophrenia. Furthermore, no impact and burden scale has been developed explicitly for use with primary caregivers based on the caregivers’ experience. ^6^ Being anchored in an exact conceptual method is needed. Development of a measure of caregivers’ impact and burden could help elucidate the biological and psychological aspects of caregiving, including quality of life, and preserving caregivers’ well-being and aptitude for care. This can help multidisciplinary treatment teams to develop new care strategies for this population. The Thai version of the Impact and Burden of Care Scale for Caregivers of Persons with Schizophrenia and Co-occurring Methamphetamine Use (TIBSCSM) was developed to assess the impact on caregivers caring for patients with schizophrenia. The objective of this study was to develop a scale and test its psychometric properties.

## Methods

### Subjects

Data were collected from the caregivers of people with schizophrenia with co-occurring methamphetamine use at psychiatric hospitals. The inclusion criteria were: (1) having a family member with a diagnosis of schizophrenia or schizoaffective disorder, according to the DSM-V criteria; ^7^ (2) being identified by persons with schizophrenia and co-occurring methamphetamine use as the primary caregiver; and (3) being 18 years of age or older.

### Procedure

Over four weeks, identified inpatients, who met the criteria of a diagnosis of schizophrenia with co-occurring methamphetamine use and were 18–60 years old, were selected. A psychiatric nurse asked them to name their primary caregiver. The researcher asked them if we could contact the primary caregiver. When they agreed, and when the caregiver met the inclusion criteria, the self-report scale was collected and completed by the researcher teams’ caregivers or interviews.

### Ethical approval

Ethical approval for the study was obtained from the Ethics Review Committee for Research Involving Human Research Participants (COA No. 284/2560). The scopes, risks, and benefits of this study for the subjects were explained. Before data collection, written consent was obtained directly from the PCPSs Participation was voluntary, and participant anonymity and confidentiality were guaranteed.

### Data collection

The data collected included the following:

1. Demographic characteristics of the primary caregivers.
2. TIBSCSM: The self-administered scale that the caregivers completed.

### Scale development

Development of the TIBSCSM occurred in two phases: a) qualitative and b) quantitative. Item generation occurred during the qualitative phase, and item reduction and the validation process happened during the quantitative phase. ^8^ The two steps involved collecting data from different subjects.

### Item generation: a qualitative approach

Item generation occurred in two steps. First, the content was derived from the questionnaire from face-to-face, semi-structured interviews performed by the researcher. Briefly, discussions addressed the impact and burden of care based on caregiver stress theory, a middle-range theory. Second, the objective was to predict caregiver stress and its outcomes from demographic characteristics, an objective burden in caregiving, stressful life events, social support, and social roles. ^9^ The determined wording of question stems and the range of response options until saturation by 30 caregivers’ interviews. The researcher performed content analysis. Third, the researcher identifies 32 questions from this interview process. These items were answered using a five-point Likert scale, defined as: 1: never/not at all; 2: rarely/a little; 3: sometimes/somewhat; 4: often/a lot; and 5: always/very much."

30 caregivers were requested to remark on any part of the scale ( *i.e.*, content, wording, response choices) that they considered inappropriate or merited improvement. Ambiguous items and those that were misinterpreted or infrequently answered were withdrawn or rephrased, leading to a preliminary scale that contained 32 items. Lastly, preliminary interviews were conducted with the caregivers to ensure that the scale was a true reflection of the caregivers’ understanding and confirmed content validity. The second round of interviews with caregivers guaranteed its face validity.

### Item reduction and validation of the TIBSCSM: a quantitative approach

The item reduction process comes from the results of statistical analyses and the steering committee’s expertise. Item response theory and classical test theory conduct by Statistical approaches. ^10^ Both metrological properties and their impact on the final scale’s content, taking into account the items’ meanings, were discussed and then removed items. The researcher retained items to produce the final version of the TIBSCSM in more robust and psychometrically sound solutions. The researcher tested for construct validity, reliability, and some aspects of external validity in the last version.

Convergent validity was evaluated by examining correlations between the TIBSCSM and two measures of caregivers’ quality of life: the Thai version of the Schizophrenia Caregiver Quality of Life Questionnaire (S-CGQoL-Thai Scale) and the Thai version of the World Health Organization Brief Quality of Life Scale (WHOQOL–BREF–THAI). Construct validity defines the construct to be assessed by the scale and measures its construct’s internal structure and the theoretical relationship of its item and subscale scores. It was evaluated using principal component factor analyses with varimax rotation ^11^ to define the number of independent items and dimensions.

## Results

### Sample characteristics

The mean age of the 142 participants was 47.1 years old (SD = 8.4). They were predominantly female (73.9%), married (63.4%), and about half had completed at least a bachelor’s degree (50.7%) and worked in agriculture (57.8%). Additionally, almost all of them (83.1%) had insufficient income, were healthy (61%), and had been the caregiver for more than five years (48%). Regarding medical history, about one-third of them (35.1%) used universal healthcare coverage, and more than half of them had no medical illness (61%) ( Table 1).

**Table 1.** Sociodemographic and clinical characteristics of caregivers of individuals with schizophrenia (n = 142).

	N	%
Caregivers		
Gender		
Male	37	26.10
Female	105	73.90
Age (years), mean ± SD 47.11 ± 8.42		
Marital status		
Single	52	36.60
Marriage	55	38.70
Widowed	12	8.50
divorced	14	9.90
separated	9	6.30
Education		
None	3	2.10
Primary/elementary education	55	38.70
Secondary education	12	8.40
Bachelor’s degree or higher	72	50.70
Employment status unemployed	6	3.90
employed	132	96.1
Income		
Sufficiency	14	9.10
Insufficiency	128	83.10
Illness		
No	94	61.04
Diabetis	2	1.30
Hypertention	7	4.55
Gastric ulcer	9	5.84
*Etc.*	6	3.90
Relationship		
Spouse	19	12.34
Child	67	43.50
Mother/Father	30	19.50
*Etc.*	26	16.90
Time of caring (year)		
1–5	68	44.16
5–10	15	9.74
>10	59	38.31
Medical payment		
Universal healthcare coverage	54	35.10
Social security service	34	22.10
Government reimbursement	25	16.20
Self-support	21	13.6
*Etc.*	8	5.20

### Construct validity and reliability

**Figure 1.**
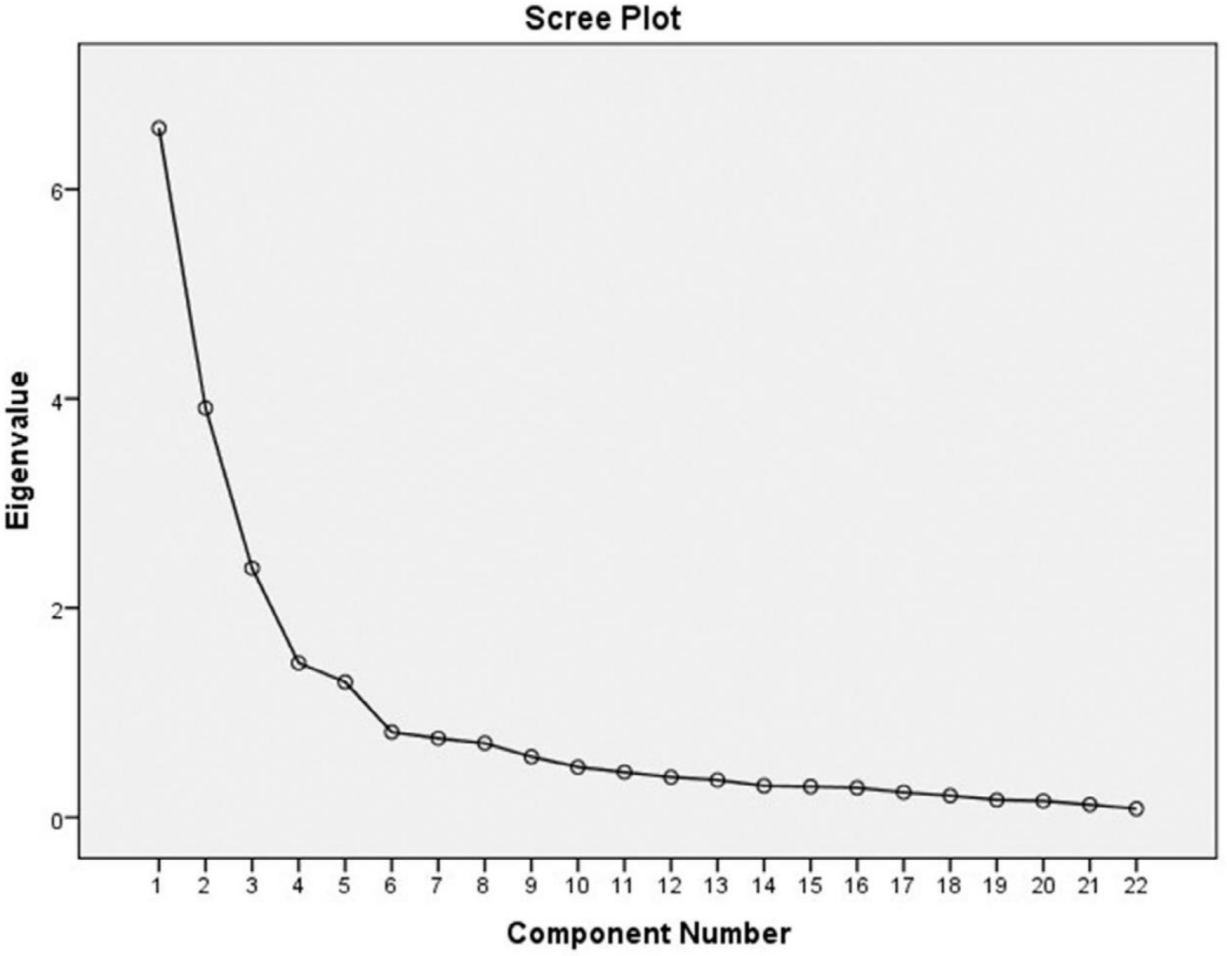
scree plot graph of TIBSCSM scale.

The TIBSCSM with 32 items showed content validity index = 1. Regarding convergent validity, the TIBSCSM had correlations with the S-CGQoL-Thai Scale and the WHOQOL–BREF–THAI. Factor analysis showed good construct validity, Kaiser–Meyer–Olkin (KMO) = 0.9, Bartlett’s Test of Sphericity, χ2 = 5248.5, df = 496, p < 0.001. Cronbach’s alpha showed high internal consistency reliability (α=0.9). The corrected item total correlation ranged 0.5–0.8. The total variance explained was 64.9%, which is excellent. Also, the extract construct of factor analysis was the following: physical function, self-esteem, role and social enjoyment, and relationship satisfaction ( Figure 1).

**Figure 2.**
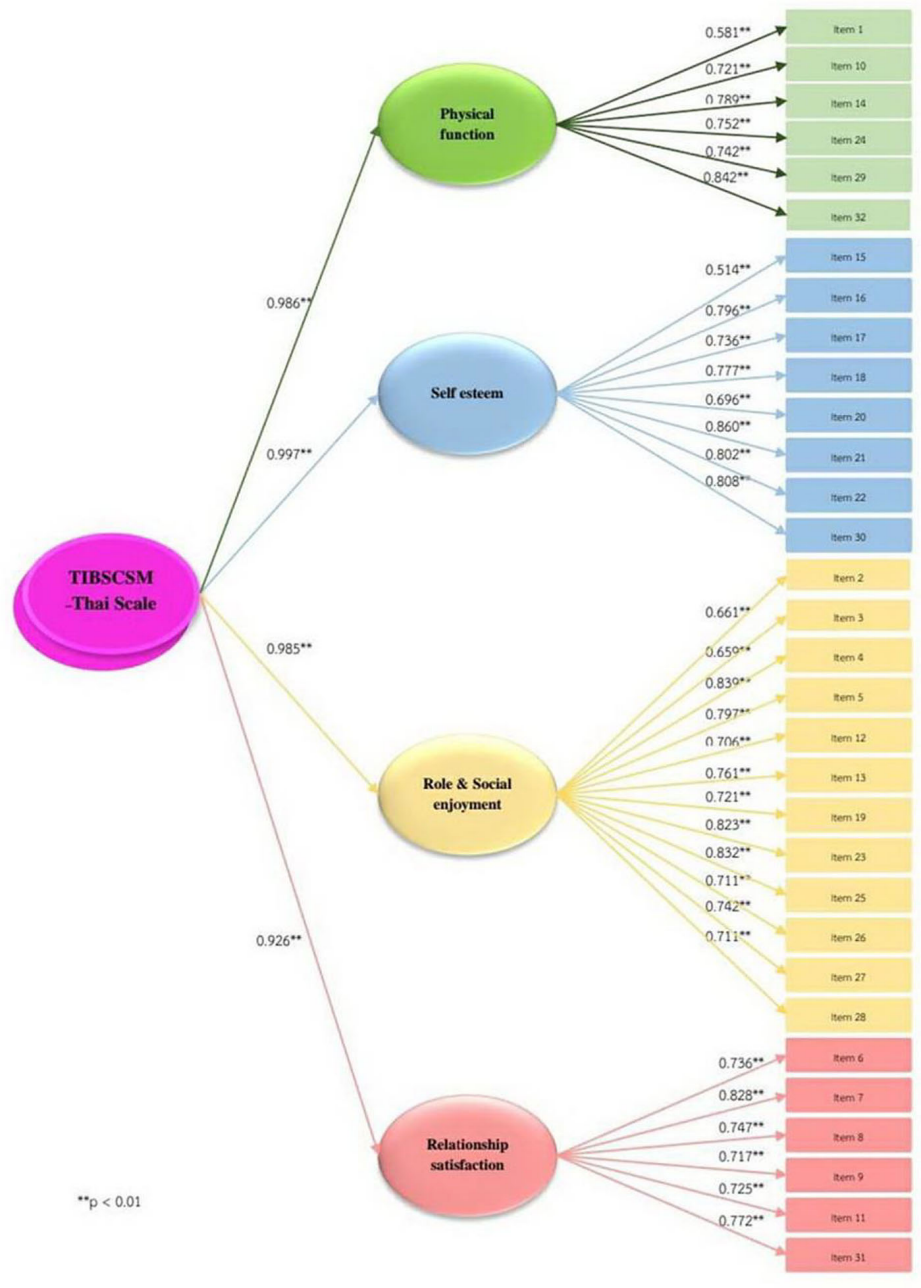
Second-order of Impact and burden care model.

Correlation testing between the 32 items found that the correlation coefficient of all 32 questions was in the range 0.3–0.8, and the correlation coefficient of the internal items of each construct were medium to high in the positive correlation with statistical significance at 0.05 ( Table 2, Figure 2).

**Table 2.** The correlation matrix of TIBSCSM.

r	IB1	IB2	IB3	IB4	IB5	IB6	IB7	IB8	IB9	IB10	IB11	IB12	IB13	IB14	IB15	IB16	IB17	IB18	IB19	IB20	IB21	IB22	IB23	IB24	IB25	IB26	IB27	IB28	IB29	IB30	IB31	IB32
IB1	1.00																															
IB2	.75	1.00																														
IB3	.68	.63	1.00																													
IB4	.60	.57	.65	1.00																												
IB5	.47	.40	.64	.80	1.00																											
IB6	.43	.47	.52	.69	.80	1.00																										
IB7	.40	.39	.57	.62	.80	.77	1.00																									
IB8	.32	.33	.58	.54	.64	.62	.71	1.00																								
IB9	.34	.35	.49	.56	.64	.64	.66	.71	1.00																							
IB10	.36	.45	.48	.45	.54	.53	.54	.57	.70	1.00																						
IB11	.48	.57	.47	.62	.56	.48	.55	.47	.60	.64	1.00																					
IB12	.43	.47	.40	.66	.58	.50	.58	.41	.48	.44	.74	1.00																				
IB13	.36	.42	.55	.66	.72	.67	.70	.50	.58	.64	.62	.67	1.00																			
IB14	.47	.48	.46	.76	.70	.64	.60	.46	.48	.50	.62	.67	.78	1.00																		
IB15	.37	.34	.52	.64	.72	.66	.70	.62	.56	.55	.43	.53	.75	.70	1.00																	
IB16	.44	.43	.51	.59	.71	.63	.69	.59	.57	.60	.52	.55	.69	.68	.72	1.00																
IB17	.52	.53	.62	.58	.63	.56	.62	.52	.62	.61	.46	.51	.64	.62	.65	.79	1.00															
IB18	.45	.45	.60	.61	.70	.66	.75	.65	.68	.55	.52	.53	.69	.66	.69	.74	.79	1.00														
IB19	.37	.47	.58	.52	.59	.52	.61	.59	.50	.58	.49	.51	.61	.58	.67	.67	.75	.74	1.00													
IB20	.33	.37	.51	.57	.57	.60	.58	.57	.56	.50	.48	.48	.55	.54	.61	.58	.57	.70	.75	1.00												
IB21	.50	.55	.57	.68	.58	.55	.59	.59	.53	.52	.64	.65	.57	.64	.65	.60	.54	.58	.71	.70	1.00											
IB22	.42	.43	.48	.65	.60	.54	.57	.57	.56	.52	.53	.61	.48	.60	.58	.61	.54	.56	.58	.57	.81	1.00										
IB23	.47	.56	.51	.61	.51	.51	.52	.54	.55	.65	.60	.53	.58	.61	.59	.63	.60	.59	.52	.47	.72	.77	1.00									
IB24	.51	.54	.46	.63	.58	.57	.55	.58	.56	.58	.56	.50	.45	.52	.49	.59	.54	.52	.40	.42	.63	.69	.81	1.00								
IB25	.52	.52	.49	.66	.60	.52	.58	.54	.57	.57	.59	.62	.49	.62	.62	.65	.59	.57	.55	.55	.73	.71	.72	.75	1.00							
IB26	.36	.42	.42	.61	.55	.45	.52	.54	.61	.50	.52	.46	.45	.48	.49	.50	.52	.60	.52	.53	.62	.55	.60	.64	.69	1.00						
IB27	.31	.40	.44	.63	.54	.42	.45	.63	.54	.56	.55	.51	.44	.53	.57	.44	.45	.48	.55	.50	.67	.67	.61	.57	.71	.75	1.00					
IB28	.26	.36	.45	.59	.49	.36	.42	.56	.44	.43	.51	.48	.41	.45	.46	.30	.30	.43	.51	.51	.73	.67	.56	.51	.60	.71	.85	1.00				
IB29	.34	.46	.45	.62	.54	.48	.45	.37	.47	.51	.55	.53	.58	.55	.43	.45	.46	.49	.48	.46	.69	.63	.63	.53	.55	.63	.65	.71	1.00			
IB30	.51	.50	.55	.66	.57	.52	.49	.50	.45	.44	.47	.60	.56	.65	.60	.60	.53	.55	.53	.53	.77	.76	.72	.63	.71	.62	.63	.68	.72	1.00		
IB31	.48	.53	.54	.59	.51	.49	.43	.55	.39	.39	.43	.47	.40	.49	.49	.52	.45	.43	.46	.46	.68	.65	.62	.60	.70	.67	.69	.71	.68	.82	1.00	
IB32	.44	.50	.49	.67	.61	.56	.64	.54	.56	.46	.60	.63	.60	.65	.57	.68	.56	.60	.51	.55	.73	.65	.66	.63	.73	.68	.60	.61	.72	.78	.77	1.00

a. Determinant = 2.503E-18.

The first-order factors loading = 0.5–0.8 and the variance of first-order factors loading were explained by a latent variable of second-order factors of the four constructs ranking 66.3% – 74.0%. Besides, factor loading of TIBSCSM, factor score, and R2 range between = 0.6–0.8, of TIBSCSM.

The second-order factors analysis of measurement model of TIBSCSM show χ2 = 325.2, df = 287, p = 0.001, RMSEA = 0, CFI0.9, TLI = 0.9, SRMR = 0 ( Table 3). This analysis shows that the scale fits with the theoretical. Factor loading: completely standardized solution of second-order factors in the high level ( Figure 1). The latent variables can explain the variance of construct of physical function, self-esteem, role and social enjoyment, and relationship satisfaction of 97.2%, 99.5%, 97.0% and 92.5%, respectively.

**Table 3.** The second-order factors analysis of measurement model of TIBSCSM.

No. of item	Constructs/items	Factor loading first-order factors	Factor loading of second-order factors		*R* ^**2**^_**total**_	*R* ^**2**^ second-order	*R* ^**2**^ first-order	
		*β*	*t*-value	*β*	*t*-value			
	**Physical function**							
1	How often do you feel that the patient asks for more help than is necessary?	0.581	11.670**	0.986	295.811**	0.337	0.328	0.009
10	How much do you feel that your health is deteriorating as a result of patient care?	0.721	20.610**	0.986	295.811**	0.519	0.505	0.014
14	How often do you feel that patients rely on you for their daily activities?	0.789	25.254**	0.986	295.811**	0.622	0.605	0.017
24	How tired are you from the care of patients?	0.752	20.256**	0.986	295.811**	0.565	0.550	0.015
29	How often do you feel that your sleep is disturbed by patient care?	0.742	18.727**	0.986	295.811**	0.551	0.535	0.016
32	How often do you feel that care for a patient affects your work? (Both paid and non-paid work)	0.842	30.997**	0.986	295.811**	0.709	0.689	0.020
	**Self-esteem**							
15	How often do you feel that you do not have enough money to take care of the patient from the rest of your expenses?	0.514	35.252**	0.997	2326.793**	0.663	0.263	0.400
16	How often do you feel that you are unable to take care of patients longer than this?	0.796	24.397**	0.997	2326.793**	0.633	0.630	0.003
17	How often do you feel that you cannot control your life because of the illness with the patient's schizophrenia?	0.736	18.729**	0.997	2326.793**	0.542	0.538	0.004
18	How often can you let other people take care of the patient instead?	0.777	23.737**	0.997	2326.793**	0.604	0.600	0.004
20	How often do you feel you can take care of more patients?	0.696	15.904**	0.997	2326.793**	0.484	0.482	0.002
21	How often do you feel you are unable to take good care of your patients?	0.860	37.871**	0.997	2326.793**	0.740	0.735	0.005
22	How difficult do you feel to care for the patient?	0.802	25.898**	0.997	2326.793**	0.644	0.639	0.005
30	How often do you feel sad from caring for patients?	0.808	26.961**	0.997	2326.793**	0.654	0.649	0.005
	**Role and Social enjoyment**							
2	How often do you feel you spend so much time with the patient that you don't have time for yourself?	0.661	15.282**	0.985	331.419**	0.437	0.424	0.013
3	How difficult is it to look after the patients and take responsibility for other things as well?	0.659	14.758**	0.985	331.419**	0.434	0.421	0.013
4	How shy do you feel about the behavior of the patient?	0.839	31.349**	0.985	331.419**	0.704	0.683	0.021
5	How frustrated are you about the behavior of the patient?	0.797	24.979**	0.985	331.419**	0.635	0.616	0.019
12	How often do you feel your social life gets worse because you have to take care of the patient?	0.706	17.886**	0.985	331.419**	0.499	0.484	0.015
13	How uncomfortable or uneasy you feel are to neglect or distance yourself from friends because of the behavior of the patient.	0.761	23.733**	0.985	331.419**	0.579	0.562	0.017
19	How often are you not sure about how to take care of the patient?	0.721	17.678**	0.985	331.419**	0.519	0.504	0.015
23	How often do you feel you have to take care of the patient alone?	0.823	28.657**	0.985	331.419**	0.677	0.657	0.020
25	How stressed do you feel from caring for patients?	0.832	30.911**	0.985	331.419**	0.692	0.672	0.020
26	How difficult do you feel when taking care of your medication?	0.711	17.089**	0.985	331.419**	0.506	0.490	0.016
27	How often do you worry that the patient's mental symptoms will relapse?	0.742	20.342**	0.985	331.419**	0.550	0.534	0.016
28	How often do you worry that the condition of the patient's schizophrenia will worsen?	0.711	17.687**	0.985	331.419**	0.506	0.490	0.016
	**Relationship satisfaction**							
6	How angry are you about the behavior of the patient?	0.736	18.687**	0.962	86.589**	0.542	0.501	0.041
7	How often do you feel that a patient's schizophrenia and substance use have a negative impact on your relationship with you?	0.828	28.049**	0.962	86.589**	0.685	0.634	0.051
8	How scared are you about what will happen in the patient's future?	0.747	21.562**	0.962	86.589**	0.559	0.516	0.043
9	How often do you feel that the patient is financially dependent on you?	0.717	20.135**	0.962	86.589**	0.514	0.476	0.038
11	How often do you feel that the patient makes you lack privacy?	0.725	18.797**	0.962	86.589**	0.525	0.486	0.039
31	How often do you experience the ups and downs of patients with schizophrenia?	0.772	21.043**	0.962	86.589**	0.596	0.552	0.044
	*χ* ^2^ = 325.273, *df* = 287, *p* = 0.060, RMSEA = 0.031, CFI = 0.993, TLI = 0.987, SRMR = 0.045							

*Note* ** *p* < 0.01.

## Discussion

The findings indicate that the TIBSCSM is psychometrically sound and well-suited for assessing caregivers’ impact and burden of care for persons with schizophrenia and methamphetamine misuse. We discuss the psychometric properties testing in the following sections.

First, the internal structure, supported by high internal consistency, confirmed that the TIBSCSM measures a multidimensional concept. Cronbach’s alpha was 0.9. This means strong correlations between items within each domain of the scale and between all items in the scale. ^12- 13^

Second, the KMO test and Bartlett test of sphericity showed the adequacy of the item correlation matrix. This reflects that the sample size was appropriate. ^11^

Third, the components’ analysis showed that the factor loadings were 0.6–0.8, which shows that the items had very high effects on the factors. The criteria for choosing an item is that the factor loading should be greater than 0.5. All questions and questions had a value of 0.5–0.8. ^11^ Additionally, the construct validity of the test was verified by factor analysis. Four components could be extracted together with the results from the scree plot graph. The parts of the four factors explained 64.9% of the variance, which shows that the 32 questions substantially explained variations in the levels of the impact and burden of care in Thai primary caregivers of people with schizophrenia who also use methamphetamine, reliably and consistently with the theory of the Caregiver Stress model. Similar to results from other empirical studies, ^14^ the findings illustrate that the predictors of family caregivers’ impact and burden care were a) physical and psychological well-being, b) dependence in performing activities of daily living, c) caring quality, d) burden and everyday life, and e) caring performance.

Additionally, the TIBSCSM, like the theory of caregiver stress, is based on the Roy Adaptation Model that identifies the caregiver’s response, perceptions, and adaptations to the stress and burden they experience in terms of their social role and how they reduce and cope with stress. Additionally, it was found that caring for people with schizophrenia often causes caregivers to perceive a high burden of care, lose energy, have anxiety and depression, and have worse overall mental health. There is an increased risk of emotional problems such as guilt, anger, and dissatisfaction. Research has found that most caregivers have emotional stress problems. ^14^ Besides, persistent requests for more help cause caregivers to become frustrated. Sometimes the emotions cannot be suppressed. Intense and negative emotions may occur.

Moreover, it has been found that many caregivers with negative cognitive processes and the inability to provide quality care may experience feelings of failure, resulting in negative perceptions of various aspects, such as having no time for family or other activities or inability to care for people with schizophrenia fully. Additionally, consistent with the caregivers’ stress theory, ^9^ effects on the caregiver include reduced physical function, feelings of low self-worth, and reduced role enjoyment and marital satisfaction, which directly affect the carers’ lives. Individual perceptions of their status in life fall under the context of the culture and system definition in a society that lives and relates to goals, expectations, society standards, and other related things. They covered physical health and mental state, degree of independence, social relations beliefs, and relationships that need to be an environment. Besides, goal burden is the most influential factor for caregivers’ stress, such as caring for hours at a time or care that extends for years. This is consistent with the personal data of more than half of the sample who have taken care of patients for more than ten years. If caregivers perceive high levels of stress, they will make ineffective responses. Additionally, the care burden may lead to depression as a direct result of the stressor may affect stress adjustment.

Besides, these responsibilities may affect other aspects of the caregiver’s life, such as interpersonal relationships. Financial conditions are defined as contextual stimuli. Social support, social roles, and life events that cause stress are life-changing conditions that challenge individuals and result in suffering. In the study sample, 83.10% of participants had incomes that were insufficient to cover their living expenses, and 45.45% had stress levels at 51–75 points.

Social support allows carers to see that they are being cared for. A high level of social support will increase the ability to cope with the burden effects of care. Additionally, social roles are defined as the caregiver’s relationship to the patient, such as being a parent or child. Most of the sample had one of these two roles, with social functions allowing caregivers to express their feelings and release their emotions, making them more likely to deal with the images and effects of care. The sample had an average age of 47 years, which can change the perspective of each person. Older caregivers have more life experiences and more opportunities to use and adjust their coping skills. ^15- 18^

## Conclusion

In conclusion, in the present paper, the findings suggested that the TIBSCSM scale has potential benefits for psychiatric and mental health care teams to assess the impact and burden of caregivers of people with schizophrenia, for both research and clinical purposes. The TIBSCSM adds exciting data oriented toward providing a more global service to those with schizophrenia and co-occurring methamphetamine use and their families. It would be significant to discover the reproducibility of the current results and their sensitivity to alteration. However, the study demonstrated that the scale has good psychometric properties. The research results deliver an innovative, valid, and essential scale that may be valuable in routine practice, clinical research, and education.

## Data availibility

Underlying data for this article have been restricted for ethical and privacy reasons. Data may be requested by contacting the authors and access will be granted to researchers and reviewers.
