# Psychometric Evaluation of the Thai Version of the Burdened Caregiver Scale for Schizophrenia and Co-occurring Methamphetamine Use: A Pilot Study

**DOI:** 10.12688/f1000research.52288.3

**Published:** 2025-04-22

**Authors:** Ek-uma Imkome

**Affiliations:** 1 Faculty of mental health and psychiatric nursing, Thammasat University, Patuntane, 12120, Thailand

**Keywords:** Schizophrenia, methamphetamine, caregivers, factor analysis, burden care.

## Abstract

**Objective:**

To develop and test the psychometric properties of the Thai version of the Impact and Burden of Care Scale for Caregivers of Persons with Schizophrenia and Co-occurring Methamphetamine Use (TIBSCSM).

**Methods:**

This pilot study involved 142 caregivers of individuals with schizophrenia and methamphetamine use. Sample adequacy was assessed using the Kaiser–Meyer–Olkin (KMO) method, while Bartlett’s test evaluated the item correlation matrix. Exploratory factor analysis (EFA) was conducted to identify underlying factors.

**Results:**

The 32-item TIBSCSM showed convergent validity correlations with two quality-of-life measures. The KMO value was 0.90, and Bartlett’s Test of Sphericity yielded χ ^2^ = 5248.5, df = 496, p < 0.001. The Content Validity Index (CVI) was 1, indicating high internal consistency reliability (α = 0.90). EFA identified four constructs: physical function, self-esteem, role and social enjoyment, and relationship satisfaction. The model exhibited strong reliability and validity, with an average variance extracted (AVE) of 0.948 and composite reliability (CR) of 0.987, accounting for 64.90% of the variance.

**Conclusion:**

The TIBSCSM scale provides valuable insights for psychiatric nurses and mental health teams to measure the impact and burden experienced by caregivers of individuals with schizophrenia and methamphetamine use. It is particularly useful for nursing, research, education, and clinical practice, especially in addressing the caregiver burdens exacerbated during the COVID-19 pandemic.

**Implication for nursing and research:**

The TIBSCSM, developed in the Thai context, can facilitate studies evaluating variations in caregiver impact across different settings.

## Introduction

Schizophrenia with co-occurring methamphetamine use is a complex and debilitating psychiatric condition that severely affects individuals across multiple domains, including physical and psychological health, social functioning, and economic stability. The chronic nature of the illness often results in a profound caregiving burden, with caregivers experiencing significant emotional distress, role confusion, and reduced quality of life. ^1, 2^ Studies indicate that caregivers frequently encounter psychosomatic symptoms, anxiety, stress, and depressive states, which, in turn, adversely affect their caregiving performance and the overall well-being of individuals with schizophrenia and substance use disorders. ^3, 4^

Caregivers employ various strategies to manage the care of individuals with schizophrenia and methamphetamine use disorder, particularly during episodes of psychosis and relapse. Strategies include fostering medication adherence, ensuring a safe environment, and providing emotional support. These efforts are critical in mitigating aggressive behaviors and sustaining continuity of care. ^5^ However, research highlights that inadequate caregiver involvement in treatment planning is associated with poor adherence to psychiatric treatment regimens, thereby emphasizing the necessity of assessing caregiving burden and its implications. ^6^

The emotional, psychological, and financial strains experienced by caregivers warrant the development of evidence-based interventions aimed at enhancing caregiver well-being. Such interventions, spearheaded by nurses and mental health professionals, could provide structured support to caregivers, improving both their psychological health and caregiving efficacy. ^7^ Despite prior studies investigating aspects of caregiving stress, there is a notable gap in research examining the specific burdens associated with schizophrenia and co-occurring methamphetamine use in primary caregivers. Furthermore, existing assessment tools do not sufficiently capture caregivers’ lived experiences in this context. ^8, 9^

Given this gap, the development of a comprehensive measurement instrument tailored to the experiences of caregivers is imperative. This study aims to develop and evaluate the psychometric properties of the Thai version of the Impact and Burden of Care Scale for Caregivers of Persons with Schizophrenia and Co-occurring Methamphetamine Use (TIBSCSM). The findings will contribute to the enhancement of multidisciplinary treatment strategies while promoting an improved quality of life for caregivers and care recipients alike.

## Methods

### Subjects

Data were collected from the caregivers of people with schizophrenia with co-occurring methamphetamine use at psychiatric hospitals. The inclusion criteria were: (1) having a family member with a diagnosis of schizophrenia or schizoaffective disorder, according to the DSM-V criteria; ^10^ (2) being identified by persons with schizophrenia and co-occurring methamphetamine use as the primary caregiver; and (3) being 18 years of age or older.

### Procedure

Over four weeks, identified inpatients, who met the criteria of a diagnosis of schizophrenia with co-occurring methamphetamine use and were 18–60 years old, were selected. A psychiatric nurse asked them to name their primary caregiver. The researcher asked them if we could contact the primary caregiver. When they agreed, and when the caregiver met the inclusion criteria, the self-report scale was collected and completed by the researcher teams’ caregivers or interviews.

### Ethical approval

Ethical approval for the study was obtained from the Ethics Review Committee for Research Involving Human Research Participants (COA No. 284/2560). The scopes, risks, and benefits of this study for the subjects were explained. Before data collection, written consent was obtained directly from the PCPSs Participation was voluntary, and participant anonymity and confidentiality were guaranteed.

### Data collection

The data collected included the following:

• Demographic characteristics of the primary caregivers.
• TIBSCSM: The caregivers completed the self-administered scale.

### Scale development

Development of the TIBSCSM occurred in two phases: a) qualitative and b) quantitative. Item generation occurred during the qualitative phase, and item reduction and the validation process happened during the quantitative phase. ^11^ The two steps involved collecting data from different subjects.

### Item generation: a qualitative approach

Item generation occurred in two steps. First, the content was derived from the questionnaire from face-to-face, semi-structured interviews performed by the researcher. Briefly, discussions addressed the impact and burden of care based on caregiver stress theory, a middle-range theory. Second, the objective was to predict caregiver stress and its outcomes from demographic characteristics, an objective burden in caregiving, stressful life events, social support, and social roles. ^7^ The determined wording of question stems and the range of response options until saturation by 30 caregivers’ interviews. The researcher performed content analysis. Third, the researcher identifies 32 questions from this interview process. These items were answered using a five-point Likert scale, defined as: 1: never/not at all; 2: rarely/a little; 3: sometimes/somewhat; 4: often/a lot; and 5: always/very much.

30 caregivers were requested to remark on any part of the scale ( *i.e.*, content, wording, response choices) that they considered inappropriate or merited improvement. Ambiguous items and those that were misinterpreted or infrequently answered were withdrawn or rephrased, leading to a preliminary scale that contained 32 items. Lastly, preliminary interviews were conducted with the caregivers to ensure that the scale was a true reflection of the caregivers’ understanding and confirmed content validity. The second round of interviews with caregivers guaranteed its face validity.

### Item reduction and validation of the TIBSCSM: a quantitative approach

The item reduction process comes from the results of statistical analyses and the steering committee’s expertise. Item response theory and classical test theory conduct by Statistical approaches. ^12^ Both metrological properties and their impact on the final scale’s content, taking into account the items’ meanings, were discussed and then removed items. The researcher retained items to produce the final version of the TIBSCSM in more robust and psychometrically sound solutions. The researcher tested for construct validity, reliability, and some aspects of external validity in the last version.

Convergent validity was evaluated by examining correlations between the TIBSCSM and two measures of caregivers’ quality of life: the Thai version of the Schizophrenia Caregiver Quality of Life Questionnaire (S-CGQoL-Thai Scale) and the Thai version of the World Health Organization Brief Quality of Life Scale (WHOQOL–BREF–THAI). Construct validity defines the construct to be assessed by the scale and measures its construct’s internal structure and the theoretical relationship of its item and subscale scores. It was evaluated using principal component factor analyses with varimax rotation ^13^ to define the number of independent items and dimensions.

## Results

### Sample characteristics

The mean age of the 142 participants was 47.1 years old (SD = 8.4). They were predominantly female (73.9%), married (63.4%), and about half had completed at least a bachelor’s degree (50.7%) and worked in agriculture (57.8%). Additionally, almost all of them (83.1%) had insufficient income, were healthy (61%), and had been the caregiver for more than five years (48%). Regarding medical history, about one-third of them (35.1%) used universal healthcare coverage, and more than half of them had no medical illness (61%) ( Table 1).

**Table 1.** Sociodemographic and clinical characteristics of caregivers of individuals with schizophrenia (n = 142).

	N	%
Caregivers		
Gender		
Male	37	26.10
Female	105	73.90
Age (years), mean ± SD 47.11 ± 8.42		
Marital status		
Single	52	36.60
Marriage	55	38.70
Widowed	12	8.50
Divorced	14	9.90
Separated	9	6.30
Education		
None	3	2.10
Primary/elementary education	55	38.70
Secondary education	12	8.40
Bachelor’s degree or higher	72	50.70
Employment status		
Unemployed	6	3.90
Employed	132	96.1
Income		
Sufficiency	14	9.10
Insufficiency	128	83.10
Illness		
No	94	61.04
Diabetis	2	1.30
Hypertention	7	4.55
Gastric ulcer	9	5.84
*Etc.*	6	3.90
Relationship		
Spouse	19	12.34
Child	67	43.50
Mother/Father	30	19.50
*Etc.*	26	16.90
Time of caring (year)		
1–5	68	44.16
5–10	15	9.74
>10	59	38.31
Medical payment		
Universal healthcare coverage	54	35.10
Social security service	34	22.10
Government reimbursement	25	16.20
Self-support	21	13.6
*Etc.*	8	5.20

### Exploratory factor analysis

The first-order factors loading = 0.5–0.8 and the variance of first-order factors loading were explained by a latent variable of second-order factors of the four constructs ranking 66.3% – 74.0%. Besides, factor loading of TIBSCSM, factor score, and R2 range between = 0.6–0.8, of TIBSCSM.

**Figure 1.**
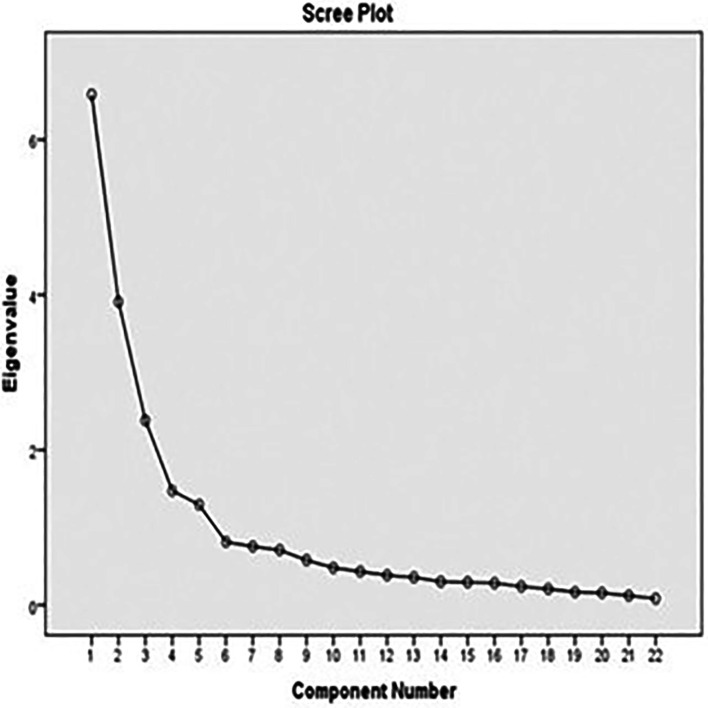
Scree plot graph of TIBSCSM scale.

The second-order factors analysis of measurement model of TIBSCSM shows factor loading completely standardized solution of second-order factors in the high level ( Figure 1). The latent variables can explain the variance of construct of physical function, self-esteem, role and social enjoyment, and relationship satisfaction of 97.2%, 99.5%, 97.0% and 92.5%, respectively. The average variance extracted (AVE) of 0.948 and composite reliability (CR) of 0.987 ( Table 3).

### Validity

Content validity: The TIBSCSM, with 32 items, showed a content validity index = 1.

Convergence validity: The TIBSCSM correlated with the S-CGQoL-Thai Scale and the WHOQOL–BREF–THAI. Factor analysis showed good construct validity, Kaiser–Meyer–Olkin (KMO) = 0.9, Bartlett’s Test of Sphericity, χ ^2^ = 5248.5, df = 496, p < 0.001. For the content validity, the scale shows CVI = 1 and S-CVI = 0.86.

### Reliability

Internal consistency: Cronbach’s alpha showed high internal consistency reliability (α = 0.9).

The corrected item total correlation ranged from 0.5–0.8. The total variance explained was 64.9%, which is excellent. Also, the extract construct of factor analysis was the following: physical function, self-esteem, role and social enjoyment, and relationship satisfaction ( Figure 1).

The mean, standard deviation, internal consistency, and reliability of various question sets: a) Physical Function (6 items: Item 1, Item 10, Item 14, Item 24, Item 29, Item 32): Mean: 52.53, S.D. =16.75, Internal Consistency: Ranges from 0.75 to 0.86, and Reliability = 0.96, b) Self-Esteem (Mastery) (9 items: Item 15, Item 16, Item 17, Item 18, Item 20, Item 21, Item 22, Item 30): Mean: 46.03, S.D. = 14.42, Internal Consistency ranges from 0.73 to 0.83, Reliability = 0.95, c) Role Enjoyment (11 items: Item 2, Item 3, Item 4, Item 5, Item 12, Item 13, Item 23, Item 25, Item 26, Item 27, Item 28): Mean = 50.04, S.D. = 13.60, Internal Consistency ranges from 0.71 to 0.84, Reliability = 0.98, d) Relationship Satisfaction (6 items: Item 6, Item 7, Item 8, Item 9, Item 11, Item 31): Mean = 49.96, S.D. = 15.62, Internal Consistency ranges from 0.67 to 0.81, and reliability = 0.86. This comprehensive data is summarized in Table 4.

**Figure 2.**
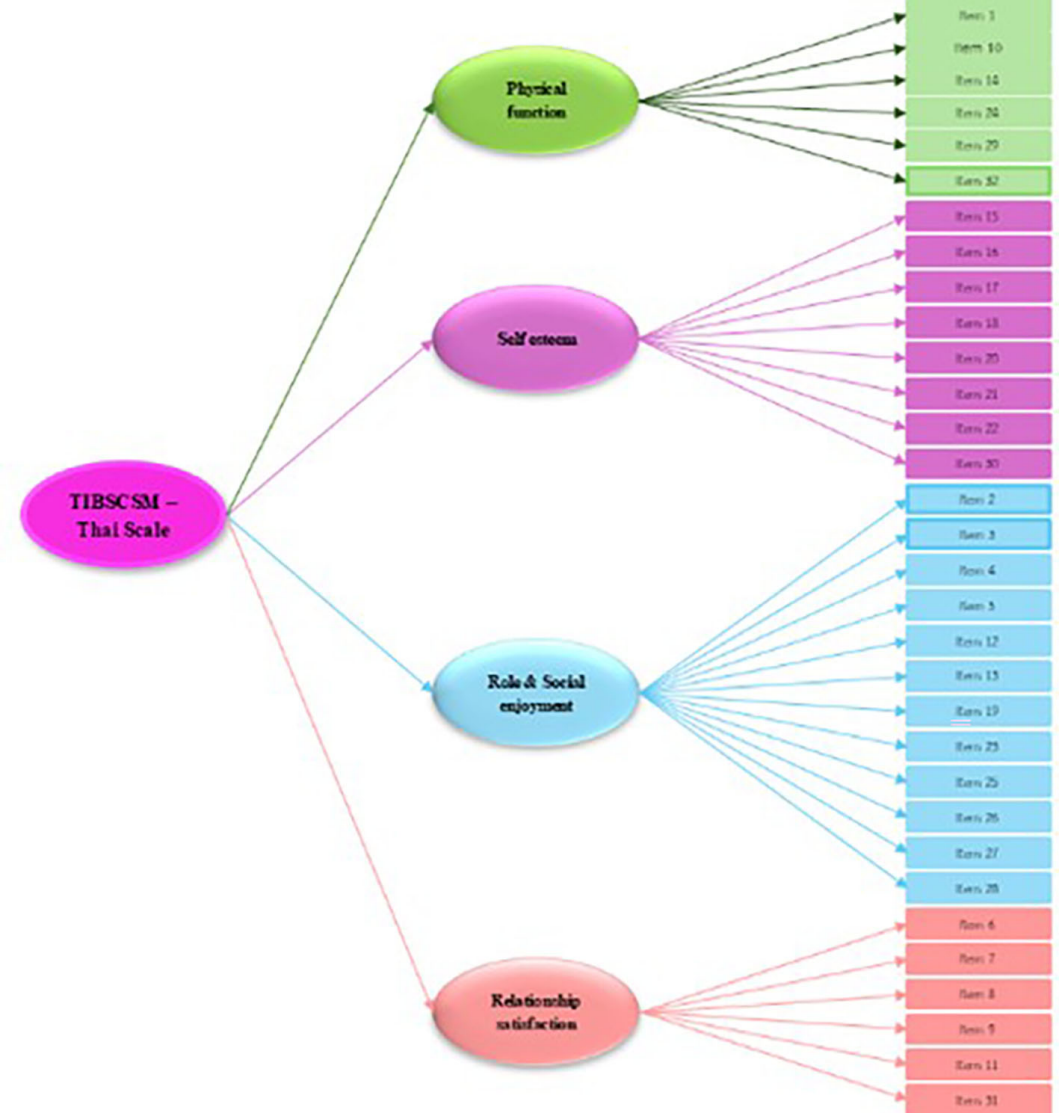
Second-order of Impact and burden care model.

Correlation testing between the 32 items found that the correlation coefficient of all 32 questions was in the range 0.3–0.8, and the correlation coefficient of the internal items of each construct were medium to high in the positive correlation with statistical significance at 0.05 ( Table 2, Figure 2).

**Table 2.** The correlation matrix of TIBSCSM.

r	IB1	IB2	IB3	IB4	IB5	IB6	IB7	IB8	IB9	IB10	IB11	IB12	IB13	IB14	IB15	IB16	IB17	IB18	IB19	IB20	IB21	IB22	IB23	IB24	IB25	IB26	IB27	IB28	IB29	IB30	IB31	IB32
IB1	1.00																															
IB2	.75	1.00																														
IB3	.68	.63	1.00																													
IB4	.60	.57	.65	1.00																												
IB5	.47	.40	.64	.80	1.00																											
IB6	.43	.47	.52	.69	.80	1.00																										
IB7	.40	.39	.57	.62	.80	.77	1.00																									
IB8	.32	.33	.58	.54	.64	.62	.71	1.00																								
IB9	.34	.35	.49	.56	.64	.64	.66	.71	1.00																							
IB10	.36	.45	.48	.45	.54	.53	.54	.57	.70	1.00																						
IB11	.48	.57	.47	.62	.56	.48	.55	.47	.60	.64	1.00																					
IB12	.43	.47	.40	.66	.58	.50	.58	.41	.48	.44	.74	1.00																				
IB13	.36	.42	.55	.66	.72	.67	.70	.50	.58	.64	.62	.67	1.00																			
IB14	.47	.48	.46	.76	.70	.64	.60	.46	.48	.50	.62	.67	.78	1.00																		
IB15	.37	.34	.52	.64	.72	.66	.70	.62	.56	.55	.43	.53	.75	.70	1.00																	
IB16	.44	.43	.51	.59	.71	.63	.69	.59	.57	.60	.52	.55	.69	.68	.72	1.00																
IB17	.52	.53	.62	.58	.63	.56	.62	.52	.62	.61	.46	.51	.64	.62	.65	.79	1.00															
IB18	.45	.45	.60	.61	.70	.66	.75	.65	.68	.55	.52	.53	.69	.66	.69	.74	.79	1.00														
IB19	.37	.47	.58	.52	.59	.52	.61	.59	.50	.58	.49	.51	.61	.58	.67	.67	.75	.74	1.00													
IB20	.33	.37	.51	.57	.57	.60	.58	.57	.56	.50	.48	.48	.55	.54	.61	.58	.57	.70	.75	1.00												
IB21	.50	.55	.57	.68	.58	.55	.59	.59	.53	.52	.64	.65	.57	.64	.65	.60	.54	.58	.71	.70	1.00											
IB22	.42	.43	.48	.65	.60	.54	.57	.57	.56	.52	.53	.61	.48	.60	.58	.61	.54	.56	.58	.57	.81	1.00										
IB23	.47	.56	.51	.61	.51	.51	.52	.54	.55	.65	.60	.53	.58	.61	.59	.63	.60	.59	.52	.47	.72	.77	1.00									
IB24	.51	.54	.46	.63	.58	.57	.55	.58	.56	.58	.56	.50	.45	.52	.49	.59	.54	.52	.40	.42	.63	.69	.81	1.00								
IB25	.52	.52	.49	.66	.60	.52	.58	.54	.57	.57	.59	.62	.49	.62	.62	.65	.59	.57	.55	.55	.73	.71	.72	.75	1.00							
IB26	.36	.42	.42	.61	.55	.45	.52	.54	.61	.50	.52	.46	.45	.48	.49	.50	.52	.60	.52	.53	.62	.55	.60	.64	.69	1.00						
IB27	.31	.40	.44	.63	.54	.42	.45	.63	.54	.56	.55	.51	.44	.53	.57	.44	.45	.48	.55	.50	.67	.67	.61	.57	.71	.75	1.00					
IB28	.26	.36	.45	.59	.49	.36	.42	.56	.44	.43	.51	.48	.41	.45	.46	.30	.30	.43	.51	.51	.73	.67	.56	.51	.60	.71	.85	1.00				
IB29	.34	.46	.45	.62	.54	.48	.45	.37	.47	.51	.55	.53	.58	.55	.43	.45	.46	.49	.48	.46	.69	.63	.63	.53	.55	.63	.65	.71	1.00			
IB30	.51	.50	.55	.66	.57	.52	.49	.50	.45	.44	.47	.60	.56	.65	.60	.60	.53	.55	.53	.53	.77	.76	.72	.63	.71	.62	.63	.68	.72	1.00		
IB31	.48	.53	.54	.59	.51	.49	.43	.55	.39	.39	.43	.47	.40	.49	.49	.52	.45	.43	.46	.46	.68	.65	.62	.60	.70	.67	.69	.71	.68	.82	1.00	
IB32	.44	.50	.49	.67	.61	.56	.64	.54	.56	.46	.60	.63	.60	.65	.57	.68	.56	.60	.51	.55	.73	.65	.66	.63	.73	.68	.60	.61	.72	.78	.77	1.00

a. Determinant = 2.503E-18.

**Table 3.** Weights of the item’s components and percentage of variance (n = 142).

	Items	Component			
		*1*	*2*	*3*	* 4*
	**Physical function**				
1	How often do you feel that the patient asks for more help than is necessary?	0.86			
10	How much do you feel that your health is deteriorating as a result of patient care?	0.76			
14	How often do you feel that patients rely on you for their daily activities?	0.81			
24	How tired are you from the care of patients?	0.75			
29	How often do you feel that your sleep is disturbed by patient care?	0.70			
32	How often do you feel that care for a patient affects your work? (Both paid and non-paid work)	0.75			
	**Self-esteem**				
15	How often do you feel that you do not have enough money to take care of the patient from the rest of your expenses?		0.75		
16	How often do you feel that you are unable to take care of patients longer than this?		0.73		
17	How often do you feel that you cannot control your life because of the illness with the patient's schizophrenia?		0.74		
18	How often can you let other people take care of the patient instead?		0.80		
20	How often do you feel you can take care of more patients?		0.67		
21	How often do you feel you are unable to take good care of your patients?		0.80		
22	How difficult do you feel to care for the patient?		0.71		
30	How often do you feel sad from caring for patients?		0.83		
	**Role and Social enjoyment**				
2	How often do you feel you spend so much time with the patient that you don't have time for yourself?			0.81	
3	How difficult is it to look after the patients and take responsibility for other things as well?			0.76	
4	How shy do you feel about the behavior of the patient?			0.76	
5	How frustrated are you about the behavior of the patient?			0.76	
12	How often do you feel your social life gets worse because you have to take care of the patient?			0.75	
13	How uncomfortable or uneasy you feel are to neglect or distance yourself from friends because of the behavior of the patient.			0.83	
19	How often are you not sure about how to take care of the patient?			0.73	
23	How often do you feel you have to take care of the patient alone?			0.74	
25	How stressed do you feel from caring for patients?			0.74	
26	How difficult do you feel when taking care of your medication?			0.71	
27	How often do you worry that the patient's mental symptoms will relapse?			0.80	
28	How often do you worry that the condition of the patient's schizophrenia will worsen?			0.84	
	**Relationship satisfaction**				
6	How angry are you about the behavior of the patient?				0.67
7	How often do you feel that a patient's schizophrenia and substance use have a negative impact on your relationship with you?				0.76
8	How scared are you about what will happen in the patient's future?				0.76
9	How often do you feel that the patient is financially dependent on you?				0.79
11	How often do you feel that the patient makes you lack privacy?				0.76
31	How often do you experience the ups and downs of patients with schizophrenia?				0.81

% of variance = 64.90%, KMO (Kaiser – Meyer – Olkin) = 0.92.

Bartlett’s Test of Sphericity χ2 = 5248.52, df =496, p < 0.001.

Average variance extracted (AVE) = 0.948, composite reliability (CR) = 0.987.

Extraction Method: Principal Component Analysis.

**Table 4.** Mean, standard deviation, internal consistency and reliability of the items (n = 142).

TIBSCSM-Thai Scale	Mean (S.D.)	IIC	Alpha ^a^
		Min–max	
1. Physical function (6 items)	52.53 (16.75)	0.75-0.86	0.96
2. Self-esteem/mastery (9 items)	46.03 (14.42)	0.73-0.83	0.95
3. Role enjoyment (11 items)	50.04 (13.60)	0.71-0.84	0.98
4. Relationship satisfaction (6 items)	49.96 (15.62)	0.67-0.81	0.86

## Discussion

This pilot study aimed to develop and evaluate the psychometric properties of the TIBSCSM, confirming its reliability and validity as an assessment tool for caregiver burden. The findings demonstrate strong internal consistency, with a Cronbach’s alpha of 0.90, indicating robust correlations between items within each dimension. ^14, 15^ This aligns with previous work on psychometric validation of caregiver burden scales, such as the study by Arslan, Yıldırım, and Ceyhan (2020), ^16^ which examined the burden scale for caregivers of older adults, demonstrating similar reliability and internal consistency.

Factor analysis results confirm that the TIBSCSM has a stable multidimensional structure, aligning with best practices for scale development and validation. ^17^ The exploratory factor analysis (EFA) identified four key dimensions—physical function, self-esteem, role and social enjoyment, and relationship satisfaction—accounting for 64.90% of the total variance. ^8^ The high KMO value (0.90) and significant Bartlett’s Test of Sphericity (χ ^2^= 5248.5, df = 496, p < 0.001) validate the adequacy of the item correlation matrix. ^13^

Further, the average variance extracted (AVE = 0.948) and composite reliability (CR = 0.987) confirm convergent validity, ensuring that the constructs measure unique dimensions while maintaining strong relationships with external assessments. ^18^ These psychometric strengths align with the methodologies outlined by Ketchen and Berg (2006) ^19^ and validate the discriminant validity of the TIBSCSM using criteria recommended by Henseler, Ringle, and Sarstedt (2014). ^20^

Compared to previously validated caregiver burden scales, such as the Schizophrenia Caregiver Quality of Life Questionnaire (S-CGQoL-Thai Scale) and the World Health Organization Brief Quality of Life Scale (WHOQOL–BREF–THAI), the TIBSCSM demonstrated strong convergent validity, reinforcing its applicability in clinical practice. ^3^ Unlike broader caregiver burden measures, which focus on general caregiving stress, the TIBSCSM is specifically designed to assess challenges related to schizophrenia and methamphetamine use, distinguishing it as a targeted assessment tool. ^4^

Arslan et al. (2020) ^16^ also emphasized the importance of psychometric rigor in caregiver burden scales, providing supporting evidence that multidimensional burden constructs can effectively differentiate specific experiences of caregivers. Similarly, research on multidimensional psychometric validation by Brunner and Süß (2005) ^21^ offers insights into the structural reliability of self-report instruments, supporting the methodological approach employed in this study.

### Implications for clinical practice and further research

This study highlights the necessity of tailoring interventions to distinct caregiver burden domains. Caregivers experiencing physical strain may benefit from structured respite care programs, while those struggling with self-esteem and emotional distress require psychological support. ^2^^,^^7^^,^^22^ The identified dimensions reflect caregivers’ diverse experiences, underscoring the need for multidisciplinary approaches to caregiving support. ^9^

Additionally, caregiver burden is intensified by financial strain, role confusion, and social stigma, reinforcing the necessity of targeted interventions to alleviate these challenges. ^23^ Previous studies indicate that caregiver stress significantly impacts patient outcomes, particularly in cases of co-occurring schizophrenia and substance use disorders. ^4^ Therefore, psychiatric nurses and mental health professionals can utilize the TIBSCSM to develop specialized training, psychoeducation programs, and policy initiatives that enhance caregiver resilience. ^5^

Further methodological refinement using variance-based structural equation modeling ^20^ could be beneficial in future studies to enhance the measurement precision of caregiver burden assessment tools. Recent advancements in factor analysis techniques, as discussed by Kock (2019), ^24^ may also contribute to improving the construct validity of psychometric instruments designed for caregiver burden assessment.

## Conclusion

This pilot study contributes to the development of standardized caregiver burden assessment tools, addressing a significant gap in psychiatric caregiving research. The TIBSCSM provides a structured framework for evaluating caregiver experiences, reinforcing the necessity of evidence-based interventions for those supporting individuals with schizophrenia and methamphetamine use. With further refinement and validation, this scale has the potential to improve clinical practice, inform policy development, and enhance caregiver well-being.

## Limitation and future directions

As a pilot study, findings should be interpreted cautiously, acknowledging the limited sample size and population specificity. While the psychometric properties of the TIBSCSM demonstrate strong preliminary validity, further cross-cultural adaptation and large-scale validation are necessary. ^6^ Future research should assess the scale’s responsiveness over time, examining its effectiveness in detecting changes in caregiver burden following structured interventions. ^25^ Additionally, longitudinal studies will be essential in confirming the stability and predictive validity of the instrument. ^11^

## Data Availability

The underlying data for this article have been restricted for ethical and privacy reasons. Data may be requested by contacting the authors and access will be granted to researchers and reviewers.
