# Supplementary material for: Psychometric Evaluation of the Thai Version of the Burdened Caregiver Scale for Schizophrenia and Co-occurring Methamphetamine Use: A Pilot Study
Source: F1000Res. 2025 Jun 2;10:484. Originally published 2021 Jun 17. [Version 4] doi: 10.12688/f1000research.52288.4 (PMC12120408; doi:10.12688/f1000research.52288.4)
Supplement: Peer review history

# Reviewer report: Reviewer response for version 4

**DOI:** 10.5256/f1000research.183111.r389327

**Published:** 2025-09-10

**Authors:** Jarurin Pitanupong

**Affiliations:** 1 Prince of Songkla University, Songkhla, Thailand

Suitable for indexing. I have no further comments to make. Thanks.

Is the work clearly and accurately presented and does it cite the current literature?

Yes

If applicable, is the statistical analysis and its interpretation appropriate?

Yes

Are all the source data underlying the results available to ensure full reproducibility?

Yes

Is the study design appropriate and is the work technically sound?

Yes

Are the conclusions drawn adequately supported by the results?

Yes

Are sufficient details of methods and analysis provided to allow replication by others?

No

Reviewer Expertise:

psychiatry and palliative

I confirm that I have read this submission and believe that I have an appropriate level of expertise to confirm that it is of an acceptable scientific standard.

# Reviewer report: Reviewer response for version 4

**DOI:** 10.5256/f1000research.183111.r389326

**Published:** 2025-06-06

**Authors:** Joko Gunawan

**Affiliations:** 1 Belitung Raya Foundation, Belitung, Indonesia

That's acceptable now if the authors remove the results of CFA using the same dataset.

Is the work clearly and accurately presented and does it cite the current literature?

Yes

If applicable, is the statistical analysis and its interpretation appropriate?

Partly

Are all the source data underlying the results available to ensure full reproducibility?

Partly

Is the study design appropriate and is the work technically sound?

Yes

Are the conclusions drawn adequately supported by the results?

Partly

Are sufficient details of methods and analysis provided to allow replication by others?

Partly

Reviewer Expertise:

Nursing

I confirm that I have read this submission and believe that I have an appropriate level of expertise to confirm that it is of an acceptable scientific standard.

# Reviewer report: Reviewer response for version 3

**DOI:** 10.5256/f1000research.155462.r385373

**Published:** 2025-05-29

**Authors:** Jarurin Pitanupong

**Affiliations:** 1 Prince of Songkla University, Songkhla, Thailand

The study presents a valuable tool for patients with schizophrenia and co-morbid substance abuse.

 However, to enhance the clarity and rigor of the manuscript, the authors should provide a clear definition of the term *primary caregiver*. The manuscript relies solely on the patient's identification of this individual without offering objective criteria. It would be beneficial to include specific attributes, such as the caregiver’s history of involvement in the patient's care, the duration and frequency of support provided, or their legal/official caregiving status, to ensure consistency and reproducibility in identifying primary caregivers across participants.

Is the work clearly and accurately presented and does it cite the current literature?

Yes

If applicable, is the statistical analysis and its interpretation appropriate?

Yes

Are all the source data underlying the results available to ensure full reproducibility?

Yes

Is the study design appropriate and is the work technically sound?

Yes

Are the conclusions drawn adequately supported by the results?

Yes

Are sufficient details of methods and analysis provided to allow replication by others?

No

Reviewer Expertise:

psychiatry and palliative

I confirm that I have read this submission and believe that I have an appropriate level of expertise to confirm that it is of an acceptable scientific standard, however I have significant reservations, as outlined above.

# Response

**Published:** 2025-05-29

**Authors:** Ek-uma Imkome

Thank you for your valuable feedback. In response, I have further clarified the definition of *primary caregiver* by incorporating additional details in the *Subjects* section, including criteria such as the caregiver’s history of involvement in the patient’s care, a minimum caregiving duration of at least one year, and their responsibility for accompanying the patient to outpatient services. These criteria align with the inclusion proposal of this study, which was approved by the Ethical Review Committee for Research Involving Human Research Participants.

 I believe these refinements will enhance the transparency, consistency, and reproducibility of caregiver identification in this version.

# Reviewer report: Reviewer response for version 2

**DOI:** 10.5256/f1000research.121367.r124128

**Published:** 2022-02-24

**Authors:** Virya Koy

**Affiliations:** 1 Chulalongkorn University, Bangkok, Thailand

**Additional comments: **

1. Goodness of Fit Statistics TIBSCSM measurement model must be included, Adjusted Goodness of Fit Index (AGFI), Goodness of Fit Index (GFI). 
2. Compare the before and after adjustment Relative Fit Index so readers will see how different before and after of the Index Values.
3. Under the figure, author must provide Goodness of Fit Statistics, including all important Relative Fit Index. 
4. I think author should not keep Item No. 1 in construct "Physical function" because the Standard Loading Weight is lower than 0.70 (.581); it is the same Item No. 1 in construct "Self-esteem (.514))"  Item No. 1 in Construct "Role and Social Enjoyment" Item No. 2 & 3 (.661 & .650 respectively) seem to be low. 

Is the work clearly and accurately presented and does it cite the current literature?

Partly

If applicable, is the statistical analysis and its interpretation appropriate?

Yes

Are all the source data underlying the results available to ensure full reproducibility?

Yes

Is the study design appropriate and is the work technically sound?

Partly

Are the conclusions drawn adequately supported by the results?

Yes

Are sufficient details of methods and analysis provided to allow replication by others?

Partly

Reviewer Expertise:

Instrument developer

I confirm that I have read this submission and believe that I have an appropriate level of expertise to confirm that it is of an acceptable scientific standard.

# Reviewer report: Reviewer response for version 2

**DOI:** 10.5256/f1000research.121367.r124129

**Published:** 2022-02-18

**Authors:** Joko Gunawan

**Affiliations:** 1 Belitung Raya Foundation, Belitung, Indonesia

The authors haven't provided any explanation about my previous comments. 

 It is still  unclear whether the authors used the same datasets/samples (142 participants) for PCA and CFA. I will not agree to use the same data for both PCA and CFA, and the authors haven't provided rationales for this issue.

Is the work clearly and accurately presented and does it cite the current literature?

Yes

If applicable, is the statistical analysis and its interpretation appropriate?

Partly

Are all the source data underlying the results available to ensure full reproducibility?

Partly

Is the study design appropriate and is the work technically sound?

Yes

Are the conclusions drawn adequately supported by the results?

Partly

Are sufficient details of methods and analysis provided to allow replication by others?

Partly

Reviewer Expertise:

Nursing

I confirm that I have read this submission and believe that I have an appropriate level of expertise to confirm that it is of an acceptable scientific standard, however I have significant reservations, as outlined above.

# Response

**Published:** 2022-03-08

**Authors:** Ek-uma Imkome

I want to clarify that the researcher can use the same dataset because of the different objectives of the study and limitations. PCA aims to define the number of independent items and dimensions.

 The EFA aims to test whether the data fit a hypothesized measurement model that this paper applies to the theory of caregiver stress, based on the Roy Adaptation Model.

 The limitation of this study is the rare group, small number of samples, vulnerability group, illegal/criminal (methamphetamine use/drug use/violence), the social stigma of patient and family caregiver, and the high stage of stress and anxiety to get involved in the research from their history of drug use and criminal, a long period of time for 2-3 years to collect the data, difficult to finding cases, and spending more time (two-years) and research budget than the estimate.

 We respect the rules and research methodology for the reviewer comments, which is a concern for further study.

# Reviewer report: Reviewer response for version 1

**DOI:** 10.5256/f1000research.55542.r99304

**Published:** 2021-11-15

**Authors:** Joko Gunawan

**Affiliations:** 1 Belitung Raya Foundation, Belitung, Indonesia

This study is important and the scale shows a good validity and reliability. Please note that my comments are not critics; just one-man opinion who needs to understand the authors' work.

- I only have one comment for this study. **It is unclear to me whether the authors used the same datasets/samples (142 participants) for PCA and CFA. Please explain it clearly.**
- If the authors used the same data, I suggest them to remove all results of CFA, just focus on EFA or PCA. **We can’t use both EFA/PCA and CFA with the same data.** The reason for this is very simple. We explore factor(s) with EFA; on the other hand, we confirm latent structure/s of the scale with CFA. So, if we try to verify the factor(s) we discovered with EFA using the same data, CFA results will most likely give good fit indices because the same data will tend to conform to the structure(s) of the scale which is discovered with EFA. 
- Or, if they have different dataset in hand, it is better to conduct another CFA with another data analysis. 

Is the work clearly and accurately presented and does it cite the current literature?

Yes

If applicable, is the statistical analysis and its interpretation appropriate?

Partly

Are all the source data underlying the results available to ensure full reproducibility?

Partly

Is the study design appropriate and is the work technically sound?

Yes

Are the conclusions drawn adequately supported by the results?

Partly

Are sufficient details of methods and analysis provided to allow replication by others?

Partly

Reviewer Expertise:

Nursing

I confirm that I have read this submission and believe that I have an appropriate level of expertise to confirm that it is of an acceptable scientific standard, however I have significant reservations, as outlined above.

# Reviewer report: Reviewer response for version 1

**DOI:** 10.5256/f1000research.55542.r93086

**Published:** 2021-09-13

**Authors:** Virya Koy

**Affiliations:** 1 Chulalongkorn University, Bangkok, Thailand

Ek-Uma Imkome investigated the topic of Development and psychometric testing of the Thai impact and burden of care scale for caregivers of persons with schizophrenia and co-occurring methamphetamine use. You are focusing on the development and assess the psychometric testing the impact and burden of care among caregivers who provide care to schizophrenia and co-occurring methamphetamine use people. The author has tried to work hard to develop a new instrument with some scientific sections. Moreover, you found an instrument composed of four constructs as follows (1) physical function, (2) self-esteem, (3) role and social enjoyment, and (4) relationship satisfaction. The manuscript is certainly well written and attractive, but I have some major concerns about the instrument development process that prevents me to endorse its acceptance at the present stage. I see the main problems with the statistics that could have led to potentially wrong results and, thus, to completely misleading conclusions.

**Title:**

- Current Title: Development and psychometric testing of the Thai impact and burden of care scale for caregivers of persons with schizophrenia and co-occurring methamphetamine use.
- Suggestion: to develop and assess the psychometric property test on Burdened Care Caregiver Scale-Thai Version for Schizophrenia and Co-occurring Methamphetamine Use.

**English:**

- English language, grammar, and punctuation needs to be revised.

**Aim:**

- The objective is not only development but includes assessment of psychometric proper also. The objective of the present study was to develop the Thai version of the Impact and Burden of Care Scale for Caregivers of Persons with Schizophrenia and Co-occurring Methamphetamine Use (TIBSCSM) and test its psychometric properties.

**Introduction:**

- The author needs to use the concept of interest consistently, e.g. 'the burden of are' and 'care burden'
- The author needs to provide readers with the definition of the concept of interest. Your concept of interest is too long, can you make it short and precise?
- “The Thai version of the Impact and Burden of Care Scale for Caregivers of Persons with Schizophrenia and Co-occurring Methamphetamine Use (TIBSCSM) was developed to assess the impact on caregivers caring for patients with schizophrenia”. It should be 'was developed and assessed'
-  The author needs to provide the reason why you want to develop this new instrument, why don’t you use the previous instrument?
- Again, you need to provide the readers with different instruments that you believe are not applicable in the Thai context.

**Methods:**

- Research design: It should be in 2 phases: first, develop what instrument? Second psychometric properties testing. This it will give readers to catch up well.
- Instrument development process:
  - Based on DeVillis (2016) there are 7 steps to conduct instrument development.
  - Authors need to explain each step therefore, readers can learn from your paper.
  - The author missed a critical and necessary step “Generating and item pool”. I can’t understand how the author gets items. The interview may not get items for new instrument development.
  - The author can use Delphi Technique or literature review to get enough items, I am not sure the interview can get items for a new instrument.
  - The most important step, authors need to conduct a pilot test for readability, comprehensiveness, administrative feasibility, and scoring of the measures, and to identify logistical management issues.
  - Missed preliminary item tryout, it leads to lack of quality instrument for the next step.
  - You need to present CVI: Item-level Content Validity Index (I-CVI) AND Content validity index (I-CVI and S-CVI/Ave).
- Sample:
  - There are 32 items, at least the author needs 160 participants, 142 is lower than the rule of instrument development.
  - How did the author recruit 30 subjects for qualitative study and 142 for quantitative approach?
  - The sampling technique is important, must be explained. 
  - A psychometric test involves multi-stage sampling process but not present it.
- Data analysis: It is a must need.

**Results:**

- Missed presenting the CVI, it is really needed for a new instrument development.
- Missed The Principal Component Analysis (PCA) explanation.
- There are 4 constructs (physical function, self-esteem, role and social enjoyment, and relationship satisfaction) but not sure they are representing to the definition of the new instrument, what is impact and burden meant? The items are not reflected about impacts and burden of care.

**Discussion:**

- Ideally, the author(s) discussed statistical analysis. Nevertheless, authors missed the discussion of each construct as the main study, authors need to explain each construct, such as physical function, self-esteem, role, social enjoyment, and relationship satisfaction. The discussion can provide readers an understanding about the researcher's perspective because the researcher is the one who knows the meaning of each construct deeply. 

**Limitations:**

- I expected authors may provide limitations, both quantitative and qualitative. 
- 142 sample are lower than the rule of a new instrument development, it needs to be discussed.

**Implication for nursing and research:**

- The implication is the vital part that the researcher has to provide the instructions on how to use this new instrument in nursing practice, and it would provide any additional ideas for the following research. For instance, the TIBSCSM is a new tool developed in the Thai context, and it may replicate the studies to evaluate variations in TIBSCSM across settings if needed.

Data needs to be re-analyzed based on the review items and generating item pool procedures to confirm results and conclusions, unless the author provides a comprehensively explanation on how to get full items before to proceed next scientific steps.

 A theoretical framework to the author needs to be addressed and guided for the instrument development process. The authors might want to consider also this for discussion.

 Lastly, I think that in the scenario generated based on DeVillis (2016) there are 7 steps to conduct instrument development.

Is the work clearly and accurately presented and does it cite the current literature?

Partly

If applicable, is the statistical analysis and its interpretation appropriate?

Yes

Are all the source data underlying the results available to ensure full reproducibility?

Yes

Is the study design appropriate and is the work technically sound?

Partly

Are the conclusions drawn adequately supported by the results?

Yes

Are sufficient details of methods and analysis provided to allow replication by others?

Partly

Reviewer Expertise:

Instrument developer

I confirm that I have read this submission and believe that I have an appropriate level of expertise to confirm that it is of an acceptable scientific standard, however I have significant reservations, as outlined above.
